# Peer-led versus routine health education for schistosomiasis knowledge improvement among primary school students in Wuhan, China

**DOI:** 10.1371/journal.pntd.0013857

**Published:** 2026-01-02

**Authors:** Yuelin Xiong, Huatang Luo, Hao Wang, Shuai Wang, Jiajing Zhang, Cong Liu

**Affiliations:** Wuhan Centers for Disease Control and Prevention, Wuhan, China; Zhejiang Wanli University, CHINA

## Abstract

**Background:**

Schistosomiasis, a neglected tropical disease (NTD), remains a public health concern in China. Health education is a fundamental intervention for its control. Even in transmission-interrupted areas like Wuhan, sustained awareness is crucial. However, recent literature on school-based interventions evaluating knowledge, attitudes and practices (KAP) among children in such areas is limited.

**Objective:**

This study aimed to evaluate and compare the effectiveness of peer-led education versus routine health education in improving schistosomiasis-related KAP among elementary students in an urban area where schistosomiasis transmission has been interrupted.

**Methods:**

A quasi-experimental school-level trial was conducted from October 2021 to January 2022 among 1013 fourth- and fifth-grade students of Yucai Hankou and Dijiao primary schools in Jiang’an district, Wuhan, China. Students were randomly assigned to two groups. Group I (n = 524) received peer-led education, while Group II (n = 489) received routine health education. Schistosomiasis-related KAP were assessed via standardized questionnaires at baseline and one month post-intervention. Statistical analyses followed the intention-to-treat principle, using multiple imputation for missing baseline data. Intervention effects were evaluated via analysis of covariance (ANCOVA) and multivariable logistic regression, adjusting for baseline scores and demographic covariates.

**Results:**

Following the intervention, both peer-led and routine health education interventions significantly improved schistosomiasis-related KAP scores among schoolchildren. For knowledge scores, both groups showed significant improvement from baseline to follow-up (within-group change: 2.93 for Group I vs. 0.98 for Group II, both *P* < 0.001). After adjusting for baseline scores, age, sex, and grade, Group I demonstrated a significantly higher adjusted mean score of 8.73 (95% CI: 8.63, 8.84) compared to Group II, which had an adjusted mean of 7.21 (95% CI: 7.10, 7.32). The adjusted mean difference (AMD) was 1.52 (95% CI: 1.37, 1.68), which was statistically significant (*P* < 0.001). For attitude scores, both groups exhibited small but statistically significant increases from baseline (within-group change: 0.20 for Group I vs. 0.21 for Group II, both *P* < 0.001). However, the between-group comparison revealed no significant difference at follow-up. For practice scores, significant within-group improvements were observed in both Group I and Group II (within-group change: 0.30 vs. 0.33, both *P* < 0.001). The adjusted mean was 3.95 (95% CI: 3.93, 3.97) in Group I and 3.97 (95% CI: 3.95, 4.00) in Group II. The AMD was -0.02 (95% CI: -0.06, 0.01), and this between-group difference was not statistically significant (*P* = 0.237). In terms of binary outcomes, the odds of achieving good knowledge were 6.04 times higher in Group I compared to Group II (aOR = 6.04, 95% CI: 4.43 to 8.24, *P* < 0.001), while no significant effects were observed on positive attitude or favorable practices between the two groups.

**Conclusion:**

Peer-led education is more effective than routine health education in improving schistosomiasis-related knowledge among primary school students in a transmission-interrupted area. Although both approaches enhanced KAP, the peer-led model demonstrated superior knowledge gains. These findings support the integration of peer-led strategies into sustainable school-based health education programmes to maintain schistosomiasis awareness and support ongoing control efforts in post-transmission settings.

## Author summary

In regions like Wuhan, China, where schistosomiasis transmission has been interrupted, sustaining knowledge and preventive behaviors through health education remains a public health priority, yet evidence on optimal strategies is limited. To address this, we conducted a quasi-experimental school-level trial among 1013 primary school students, comparing a peer-led education approach to routine health education. Our study found that while both interventions successfully improved students’ attitudes and practices, peer-led education was significantly more effective at enhancing knowledge scores. These results demonstrate that participatory, peer-led methods are superior for knowledge retention and should be integrated into sustainable control programs in post-elimination settings to maintain protective behaviors and prevent resurgence among key at-risk groups like schoolchildren.

## 1. Introduction

Schistosomiasis remains a significant public health challenge [1,2], particularly in endemic regions like China, where *Schistosoma japonicum* persists due to zoonotic transmission and complex ecologies [3,4]. The zoonotic nature of *S. japonicum*, involving bovines and rodents as reservoir hosts, underscores that its sustainable control necessitates a One Health approach—integrating human, animal, and environmental health strategies [5]. Wuhan, the capital city of Hubei Province, a historically endemic area located in the middle and lower Yangtze River basin, achieved schistosomiasis transmission interruption by 2017 through comprehensive control measures, including praziquantel-based chemotherapy, environmental modification, molluscicide application, and health education [6]. However, the persistence of snail habitats continues to pose a rebound risk [7,8], underscoring the need for sustained interventions aligned with the One Health perspective to meet the national goal of elimination by 2030 [9].

In this post-elimination phase, health education is paramount to prevent resurgence by maintaining protective behaviors among at-risk populations [10,11]. While routine health education has been a cornerstone of China’s schistosomiasis control programme [12], its long-term effectiveness in maintaining awareness in transmission - interrupted zones can be limited [13]. Evidence suggests that school-based education is especially critical, as children are both a vulnerable group and key agents for disseminating health knowledge within their communities [14]. The knowledge–attitude–practice (KAP) model provides a robust framework for evaluating such educational interventions, positing that increased knowledge fosters positive attitudes and, ultimately, sustains preventive practices [15,16]. Peer-led education, rooted in social learning theory [17], offers a promising alternative by leveraging trust and relatability among students to enhance knowledge assimilation and behavioral change [18–20]. This model has proven effective in various health promotion domains [18–20] yet remains critically underutilized and unevaluated in the context of schistosomiasis control in China.

To address this gap, this study employed a quasi-experimental school-level trial to compare the effectiveness of a structured, peer-led education intervention against routine health education in improving schistosomiasis-related knowledge, attitudes, and practices (KAP) among primary school students in Wuhan, China, over a 3-month period. We hypothesized that the peer-led education approach would result in significantly greater improvements in schistosomiasis-related knowledge, attitudes, and practices (KAP) compared to the routine health education approach. Specifically, we posited that peer-led education would be superior in improving schistosomiasis-related knowledge (primary outcome), as well as in fostering more positive attitudes and promoting better preventive practices (secondary outcomes). While this school-based intervention primarily targets the human behavioral component of the One Health spectrum, an effective education for sustained vigilance must also foster an understanding of the disease’s zoonotic and environmental dimensions. This study therefore also serves as a foundational step towards exploring how core One Health principles can be integrated into future health education curricula for comprehensive schistosomiasis control in post-elimination settings.

## 2. Materials and methods

### 2.1. Ethics statement

The current study was approved by the Research Ethics Committee of Wuhan Centers for Disease Control and Prevention (Ethical Code: WHCDCIRB-K-2021010). Written informed consent was obtained from the parents or legal guardians of all child participants. Additionally, written assent was obtained from each participating child.

### 2.2. Study area and population

**Fig 1:**
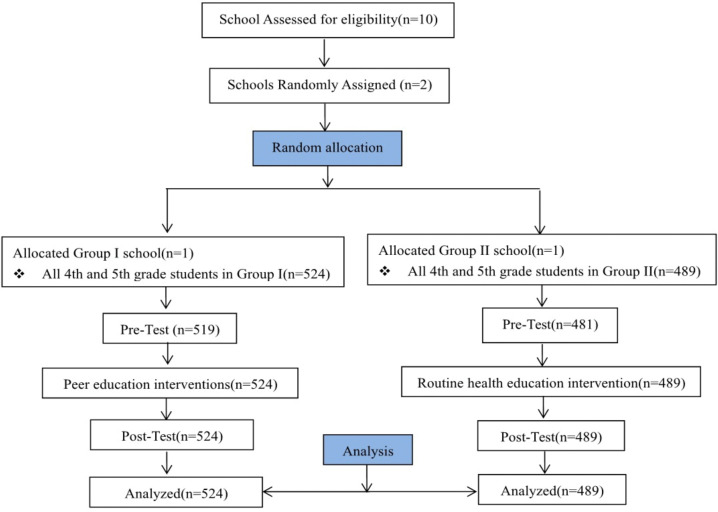
Flowchart of school allocation, intervention groups, and analysis.

This quasi-experimental study with school-level random allocation was conducted at Yucai Hankou Primary School and Dijiao Primary School in Jiang’an district of Wuhan, Hubei Province, China, from October 2021 to January 2022. The two schools were randomly assigned to one of two intervention arms: Yucai Hankou Primary School was assigned to Group I (peer-led education, n = 524) and Dijiao Primary School was assigned to Group II (routine education, n = 489) (Fig 1). Only one school per arm was feasible because of administrative constraints, consequently, the study is best described as a two-school parallel-group trial with random allocation at the cluster level rather than a conventional cluster randomized controlled trial. All fourth and fifth-grade students from both schools were invited to participate. Although no formal a priori sample size calculation was performed, this convenience sampling approach aimed to maximize statistical power within the project’s logistical constraints. The final enrolled sample of 1013 participants provided substantial power, with a post-hoc analysis indicating > 90% power (two-sided ɑ = 0.05) to detect a small effect size (Cohen’s d ≈ 0.2) in the primary knowledge score, which is considered to be of educational importance.

Jiang’an district, an urban center in northwestern Wuhan adjacent to the Yangtze River, consisting of 16 subdistricts and 1 community. The total population at the commencement of the study was 965300, with approximately 1% engaged in either agriculture or fishing. It has a historical burden of schistosomiasis caused by *Schistosoma japonicum*. From 1998 to 2015, integrated control measures emphasizing environmental improvements, including snail habitat modification through water resource projects, achieved transmission interruption. However, living Oncomelania hupensis snails—intermediate hosts essential for the schistosome life cycle—persist annually in Yangtze River marshlands within Wuhan. Currently, while tap water supplied by Wuhan Water Group Company Limited ensures safe drinking water, the persistence of snails necessitates ongoing vigilance against exposure during recreational activities, such as swimming, fishing, and playing in water.

**Fig 2:**
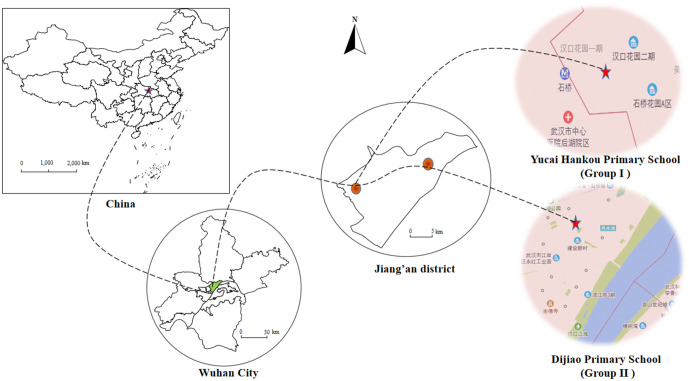
Map of the study area in Jiang’an District, Wuhan, China. This map was adapted from a standard map (Review Number: GS (2019)1652) obtained from the Standard Map Service (http://bzdt.ch.mnr.gov.cn/) of the National Bureau of Surveying and Mapping Geographic Information. The base map has not been modified.

Prior to randomization, the two schools were assessed for key characteristics to ensure a reasonable degree of comparability. Both were well-resourced public institutions in Jiang’an district with qualified teaching staff, comprising approximately 80 teachers and over 1500 students. Although located at a considerable distance apart, both student populations were known to frequent the Jiang’an section of the Yangtze River marshland, a preferred recreational destination that still harbors snail populations. This area still has snail distribution and was selected for the study due to its representative transmission-interrupted marshland ecology, history as an urban endemic area for schistosomiasis, and established institutional cooperation (Fig 2).

Baseline data on schistosomiasis prevention and control knowledge, attitudes, and practices (KAP) were collected via a KAP questionnaire in October 2021. School-based educational interventions, implemented in two distinct formats, commenced in November 2021. After three months of intervention, the effectiveness of the education was assessed using the same KAP questionnaire.

### 2.3. Questionnaire survey

The KAP questionnaire, administered in Chinese, was adapted from validated instruments used in previous schistosomiasis studies conducted in China [21,22]. It comprised 10 key items on knowledge of pathology, symptoms, cause, transmission routes, prevention and treatment of schistosomiasis, with specific questions on core concepts such as the role of praziquantel as the primary treatment drug and the risk of defecating in freshwater sources. The questionnaire also included 3 items on attitudes and 3 items on behaviors towards schistosomiasis prevention and control.

Each item on knowledge section had four responses: one correct response, two incorrect responses and one “Don’t know” response to avoid guessing. Correct responses scored one point, incorrect or “Don’t know” responses scored zero. The total knowledge score was the sum of all correct answers, ranging from 0 to 10.

For each of the three attitude items, responses indicating a positive or proactive disposition were scored 1 point. Responses indicating a negative, passive, or uncertain disposition were scored 0. The total attitude score was the sum of the three items, ranging from 0 to 3.

Each behavior item was scored based on the safety and proactivity of the practice: for Item 1, the response “Never” was scored 2 points, “Occasionally” was scored 1 point, and “Frequently” was scored 0. For Item 2, the response “Yes” was scored 1 point, and “No” was scored 0. For Item 3, the response “Go immediately” was scored 1 point, while “Don’t want to go” and “Don’t know what to do” were scored 0.The total behavior score was the sum of the three items, ranging from 0 to 4.

Closed-ended questions were used for all sections. To ensure data quality and instrument validity, the questionnaire was pre-tested for clarity, comprehensibility, and appropriate completion time with a small group of students from a non-participating school prior to the study commencement. Furthermore, all researchers involved in data collection underwent standardized training to ensure a consistent and thorough understanding of every questionnaire item and the data collection procedure.

The questionnaire was administered both before and one month after the intervention. Completion time averaged 15–20 minutes. Prior to baseline data collection, all researchers underwent standardized training to ensure thorough familiarity with every questionnaire item. Completed questionnaires were collected immediately by the researchers.

### 2.4. Study interventions

This study compared two structured educational interventions. Both Group I and Group II received three monthly 45-minute sessions covering the essential knowledge of schistosomiasis transmission, symptoms, harmfulness, and control strategies.

For Group I, the intervention was implemented through a peer-led approach. First, four to five potential peer educators were selected per class through anonymous voting based on leadership and communication skills. Two weeks prior to program implementation, all the selected students completed a standardized 15-hour training program delivered by school teachers with support from Wuhan CDC experts. The training covered both core schistosomiasis knowledge and participatory facilitation techniques, including role-playing, group discussion, brainstorming, and questions-and-answers, the use of multimedia device like flip charts, the preparation of slides and PowerPoint presentations. A subsequent 3-day rehearsal session ensured message accuracy. During implementation, participants were divided into 18 subgroups. Each subgroup underwent sessions facilitated by a team of three trained peer educators in the school’s multimedia classrooms, utilizing interactive methods like discussions, role-plays, and animated videos. Project researchers and head teachers oversaw the coordination (Table 1).

**Table 1:** Summary of Training Content for Peer Educators.

Training Module	Key Content	Methods & Techniques
1. Core Schistosomiasis Knowledge	Transmission routes (life cycle of schistosomiasis caused by *Schistosoma japonicum*)	Interactive lecture, Q&A session with Wuhan CDC experts
	Symptoms and health impacts	
	Prevention and control strategies	
	Local relevance (Wuhan marshlands)	
2.Participatory Facilitation Skills	Principles of peer education	Demonstration, practice sessions, feedback
	How to lead group discussions	
	Conducting role-plays and brainstorming sessions	
	Effective communication and active listening	
3.Educational Material & Presentation Skills	Using flip charts and preparing PowerPoint slides	Hands-on practice, rehearsal with feedback from teachers
	Key message delivery and simplifying complex information	
	Time management during sessions	

**Fig 3:**
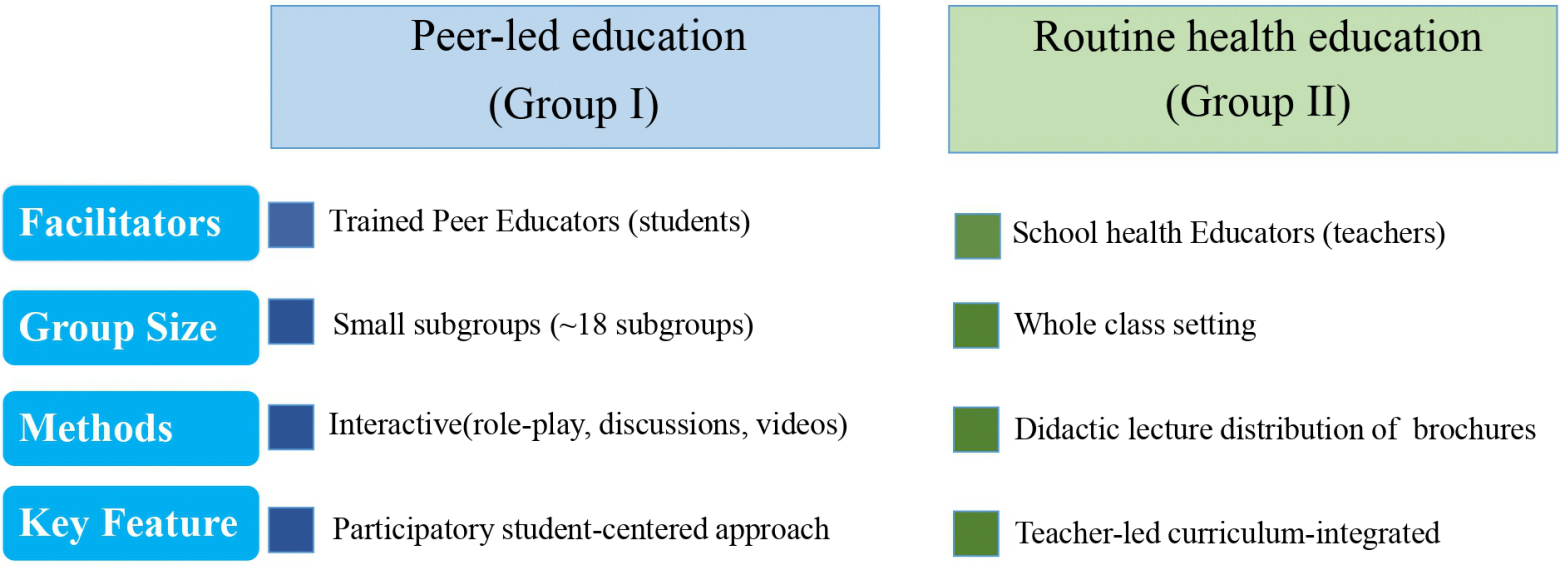
Schematic diagram comparing the key characteristics of the peer-led and routine health education intervention.

In contrast, Group II received routine health education integrated into the standard curriculum. School health educators, who first received standardized training prior to the intervention, delivered the three sessions using conventional didactic methods. The schistosomiasis content was seamlessly incorporated into the existing curriculum without disrupting its core objectives, and was supplemented by distribution of prevention brochures provided by the researchers (Fig 3).

For their efforts, all participants in the study received small appreciation gifts (a pen, notebook, and pencil case) upon study completion.

### 2.5. Statistical analysis

After collecting and screening the questionnaires, two researchers independently performed double-data entry using Epi Data 3.1. All statistical analyses were conducted using SAS 9.4 (SAS Institute, Cary, North Carolina, USA). The primary analysis followed the intention-to-treat (ITT) principle, with all 1013 randomly assigned participants analyzed according to their original group assignments for assessing intervention effectiveness. Baseline KAP data were missing for 13 participants. To adhere to the ITT principle, multiple imputation (MI) using the fully conditional specification (FCS) method was employed to create five complete datasets for the baseline knowledge, attitude and practice scores. The imputation model included the corresponding post-intervention score (e.g., baseline knowledge score was imputed using post-intervention knowledge score), group, age, sex, and grade. Results from the imputed datasets were pooled according to Rubin’s rules.

To evaluate the intervention effect on each dimension, separate multivariable analyses of covariance (ANCOVA) were performed for knowledge, attitude, and practice scores using PROC MIXED. Each model included post-intervention score (e.g., post-intervention knowledge score) as the dependent variable, group as a fixed factor, and the corresponding baseline score, age, sex, and grade as covariates. Adjusted mean differences (AMD) between groups and their 95% CIs are reported for each outcome. Separately, paired *t*-tests were used to assess the changes in knowledge, attitude, and practice scores from baseline to follow-up within each group.

For the logistic regression analysis, the total scores for knowledge, attitude, and practices were dichotomized into binary outcomes. Based on the pre-specified criteria, a participant was classified as having good knowledge if the knowledge score was ≥ 8 points, having a positive attitude if the attitude score was 3, and having favorable practices if the practice score was 4. These binary variables were used as dependent variables in separate logistic regression models. Multivariable logistic regression was fitted for each outcome, adjusting for its baseline binary value, sex, age, and grade. Odds ratios (ORs) with 95% CIs are reported. The significance level for all statistical tests was set at *P *< 0.05. A Bonferroni correction was applied to the analysis of the three primary outcomes (knowledge, attitude, and practice scores) to maintain the family-wise error rate. The significance level for between-group comparisons of these outcomes was therefore set at *P *< 0.0167 (0.05/3).

## 3. Results

### 3.1. Demographic characteristics of the population

A total of 1013 participants were enrolled in this study (Group I: n = 524, Group II:n = 489). Among them, 1000 participants (Group I: n = 519, Group II: n = 481) completed both the baseline and one month follow-up questionnaires, yielding a response rate 98.72% (Fig 1). The mean age of all participants was 10.21 ± 0.61 years. As shown in Table 2, there were no statistically significant differences in demographic characteristics (age, sex, or grade) between the two groups at baseline (*P* > 0.05).

**Table 2:** Baseline demographic characteristics of the study participants.

Variables		Group I	Group II	*P-*value^a^
		n = 524	n = 489	
Age (year, mean ± SD)		10.22 ± 0.60	10.20 ± 0.62	0.695^b^
Sex, *n* (%)	Female	247 (47.14)	230 (47.03)	0.974
	Male	277 (52.86)	259 (52.97)	
Grade, *n* (%)	Fourth	263 (50.19)	233 (47.65)	0.419
	Fifth	261 (49.81)	256 (52.35)	
Total, *n* (%)		524 (100.00)	489 (100.00)	

a: Independent *t*-test or Chi-square test between Group I and Group II; b:*P*-value for age derived from independent *t*-test; others from Chi-square test. SD: Standard Deviation.

### 3.2. Intervention effects on knowledge, attitude, and practice scores

The effects of the interventions were evaluated in line with the pre-specified analysis plan, using analysis of covariance (ANCOVA) to compare adjusted mean scores between groups at follow-up, while controlling for baseline scores and demographic covariates. The effects are summarized in Table 3.

**Table 3:** Within-Group changes and between-Group differences in knowledge, attitude, and practice scores.

Outcome Measure	Group	N	Baseline Score, Mean (SD)	Follow-up Score, Mean (SD)	Within-Group Change (95% CI)^a^	*P* -value^a^	Adjusted Mean at Follow-up (95% CI)^b^	AMD (95% CI)^b^	*P*-value^b^
Knowledge Score	Group I	524	5.77 (0.07)	8.70 (0.06)	2.93 (2.76,3.11)	<0.001	8.73 (8.63,8.84)	1.52 (1.37,1.68)	<0.001
	Group II	489	6.27 (0.10)	7.25 (0.06)	0.98 (0.76,1.19)	<0.001	7.21 (7.10,7.32)	Reference	
Attitude Score	Group I	524	2.77 (0.03)	2.97 (0.01)	0.20 (0.15,0.26)	<0.001	2.97 (2.95,2.99)	-0.01 (-0.03,0.01)	0.363
	Group II	489	2.77 (0.03)	2.98 (0.01)	0.21 (0.16,0.26)	<0.001	2.98 (2.96,3.00)	Reference	
Practice Score	Group I	524	3.65 (0.03)	3.95 (0.01)	0.30 (0.23,0.36)	<0.001	3.95 (3.93,3.97)	-0.02 (-0.06,0.01)	0.237
	Group II	489	3.64 (0.03)	3.97 (0.01)	0.33 (0.26,0.40)	<0.001	3.97 (3.95,4.00)	Reference	

a:Based on paired *t*-test, comparing scores from baseline to follow-up within each group; b:Based on analysis of covariance (ANCOVA), adjusting for the corresponding baseline score, age, sex, and grade. AMD: Adjusted Mean Difference; SD: Standard Deviation; CI: Confidence Interval.

**Fig 4:**
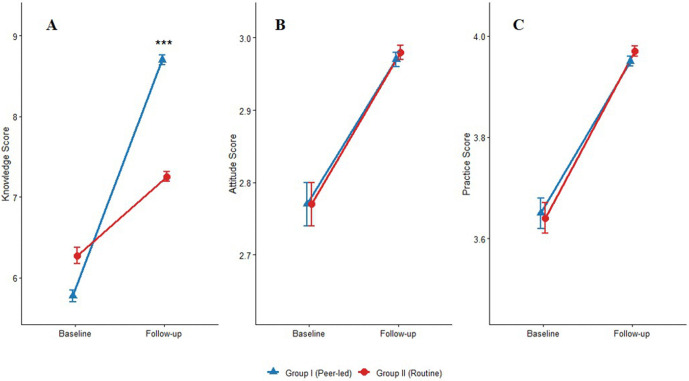
Changes in schistosomiasis-related (A) knowledge, (B) attitude, and (C) practice scores following educational interventions. Data are presented as mean ± standard error. ***P < 0.001 for between-group difference at follow-up (ANCOVA, adjusted for baseline scores and demographics). No significant between-group differences were observed for attitude or practice scores.

In terms of knowledge scores, both groups showed significant improvement from baseline to follow-up (within-group change: 2.93 for Group I vs. 0.98 for Group II, both *P* < 0.001). After adjusting for baseline scores and other covariates, Group I demonstrated a significantly higher adjusted mean score of 8.73 (95% CI: 8.63, 8.84) compared to Group II, which had an adjusted mean of 7.21 (95% CI: 7.10, 7.32). The adjusted mean difference (AMD) was 1.52 (95% CI: 1.37, 1.68), which was statistically significant after Bonferroni correction (*P* < 0.001). As clearly illustrated in Fig 4A, Group I showed substantially greater improvement in knowledge scores compared to Group II.

With regard to attitude scores, both groups exhibited small but statistically significant increases from baseline (within-group change: 0.20 for Group I vs. 0.21 for Group II, both *P* < 0.001). However, the between-group comparison revealed no significant difference at follow-up. The adjusted mean for Group I was 2.97 (95% CI: 2.95, 2.99), while Group II had an adjusted mean of 2.98 (95% CI: 2.96, 3.00). The AMD was -0.01 (95% CI: -0.03, 0.01), which was not statistically significant (*P* = 0.363, not significant at the Bonferroni-adjusted level of 0.0167). Visual inspection of Fig 4B confirms the parallel and minimal changes in attitude scores for both intervention groups.

Similarly, for practice scores, significant within-group improvements were observed in both Group I and Group II (within-group change: 0.30 vs. 0.33, both *P* < 0.001). The adjusted mean was 3.95 (95% CI: 3.93, 3.97) in Group I and 3.97 (95% CI: 3.95, 4.00) in Group II. The AMD was -0.02 (95% CI: -0.06, 0.01), and this between-group difference was not statistically significant (*P* = 0.237, not significant at the Bonferroni-adjusted level of 0.0167). Fig 4C demonstrates nearly identical patterns of practice score improvement across both educational approaches.

### 3.3. Binary outcomes: good Knowledge, positive attitude, and favorable practices

Consistent with the statistical plan, multivariable logistic regression was employed to assess the intervention effects on the dichotomized outcomes of good knowledge, positive attitude, and favorable practices, after adjusting for their baseline status and other covariates. The results are presented in Table 4. The Group I significantly increased the odds of achieving good knowledge, but no significant effects were observed on positive attitude or favorable practices compared to the Group II.

**Table 4:** Logistic regression analysis for binary outcomes of knowledge, attitude, and practices.

Outcome variables	Group	Unadjusted OR (95% CI)	*P*-value	Adjusted OR (aOR) (95% CI)^a^	*P*-value
Good knowledge^b^	Group I	5.04 (3.80,6.67)	<0.001	6.04 (4.43,8.24)	<0.001
	Group II	Reference		Reference	
Positive attitude^c^	Group I	0.82 (0.36,1.89)	0.642	0.78 (0.33,1.81)	0.559
	Group II	Reference		Reference	
Favorable practices^d^	Group I	0.71 (0.32,1.59)	0.404	0.75 (0.33,1.70)	0.490
	Group II	Reference		Reference	

a: Adjusted for the baseline binary status of the outcome, sex, age, and grade; b: Good knowledge was defined as a knowledge score ≥ 8 points; c: Positive attitude was defined as an attitude score of 3; d:Favorable practices was defined as a practice score of 4. OR: Odds Ratio; CI: Confidence Interval.

For good knowledge, the unadjusted model indicated that the odds of having good knowledge were 3.85 times higher in the Group I than in the control group (OR = 5.04 95% CI: 3.80 to 6.67, *P* < 0.001). After adjusting for baseline knowledge level, sex, age, and grade, this significant association remained robust. Specifically, participants in the Group I had 6.04 times higher odds of possessing good knowledge compared to those in the Group II (aOR = 6.04, 95% CI: 4.43 to 8.24, *P* < 0.001, significant after Bonferroni correction).

For positive attitude, no statistically significant differences were found between the groups in either the unadjusted model (OR = 0.82, 95% CI: 0.36 to 1.89, *P* = 0.642) or the model adjusted for baseline attitude, sex, age, and grade (aOR = 0.78, 95% CI: 0.33 to 1.81, *P* = 0.559, not significant after Bonferroni correction).

Similarly, for favorable practices, no significant between-group differences were observed. The unadjusted odds ratio was 0.71 (95% CI: 0.32 to 1.59, *P* = 0.404), and the adjusted odds ratio was 0.75 (95% CI: 0.33 to 1.70, *P* = 0.490, not significant after Bonferroni correction).

## 4. Discussion

This quasi-experimental study with school-level random allocation provides clear evidence that both peer-led and routine health education are effective in improving schistosomiasis-related knowledge, attitudes, and practices among primary school students in a transmission-interrupted area of Wuhan, China. The central finding, however, is the marked superiority of the peer-led approach in enhancing knowledge acquisition, as demonstrated by the significant adjusted mean difference in post-intervention knowledge scores and the substantially higher odds of students achieving good knowledge compared to those receiving routine education. This outcome aligns with the principles of social learning theory [17], wherein peer educators act as relatable and credible models, facilitating a more engaging and participatory learning environment that promotes deeper information processing and retention. The efficacy of peer-led strategies for knowledge dissemination is well-documented in other public health domains, such as HIV prevention [18,20], underscoring the power of leveraging shared social identities for educational purposes.

Despite this pronounced effect on knowledge, the study revealed a critical dissociation between knowledge gains and changes in attitudes or practices, with no statistically significant differences observed between the two intervention groups for these latter outcomes. This discrepancy underscores the complexity of the knowledge-attitude-practice continuum. The high baseline scores for attitudes and practices likely indicate a ceiling effect [23], attributable to the long-standing success of China’s national schistosomiasis control programme, which has already instilled a strong foundational level of awareness and prophylactic behavior in this population. Consequently, the marginal room for further improvement through short-term, school-based interventions was inherently limited. Furthermore, the transformation of new knowledge into solidified attitudes and sustained behavioral practices is a complex process influenced by a myriad of factors beyond the classroom, including parental influence, community norms, and environmental cues [24,25]. The three-month duration of our intervention, while sufficient for knowledge transfer, may have been inadequate to catalyze a measurable shift in these more deeply ingrained dimensions. This interpretation is consistent with literature suggesting that behavioral changes often require longer-term, multi-faceted interventions [26,27]. Furthermore, our findings can be viewed through the lens of the One Health paradigm, which emphasizes the interconnectedness of human, animal, and environmental health for zoonotic disease control [5]. While our school-based intervention effectively targeted the human behavior component, the persistence of *S. japonicum* in zoonotic reservoirs and snail habitats in the region underscores that educational strategies alone cannot address the full transmission cycle. The observed ceiling effect in attitudes and practices may thus also reflect a pragmatic public understanding that individual vigilance must operate in tandem with broader veterinary and environmental management strategies to sustainably mitigate risk [9].

Our findings have direct implications for sustainable schistosomiasis control in post-transmission settings. Peer-led education emerges as a powerful, evidence-based strategy for rapidly enhancing knowledge reserves within school populations. Health policymakers should consider its integration into existing school health programmes as a complementary strategy to routine education. However, the decision to implement peer-led programmes must be balanced against considerations of resource allocation and cost-effectiveness. The recruitment, training, and supervision of peer educators demand significant investment of time and effort. In resource-constrained settings where maintaining already-high levels of awareness is the primary goal, routine health education might represent a more feasible and scalable option. Future economic evaluations are warranted to guide this decision-making process. Beyond economic considerations, the content of such educational programmes — whether peer-led or routine — could be enhanced by integrating core One Health principles. For instance, future curricula could incorporate modules on the role of livestock and other animal reservoirs in the transmission cycle, and the critical importance of sanitation in breaking the environmental transmission pathway. Educating students on these interconnected elements would foster a more holistic understanding of disease control, empowering them as informed advocates for the multi-sectoral strategies essential for achieving and sustaining elimination.

Several limitations of our study should be acknowledged. First, the three-month follow-up period restricts our ability to assess the long-term sustainability of the knowledge gains and the potential for delayed attitude or practice changes. Second, a key methodological limitation pertains to the study design. Although the two schools were randomly assigned to intervention arms, the use of only two clusters (one school per arm) limits the robustness of our study as a conventional cluster randomized controlled trial (cRCT). This design does not allow for statistical control of intra-cluster correlation at the school level, and the observed effects could potentially be influenced by unmeasured school-level confounders. Therefore, the internal validity of the causal inference between the intervention and the outcomes should be interpreted with caution. Third, the participants were recruited from only two schools in one urban district of Wuhan. This, along with the relatively short three-month follow-up period, limits the generalizability of our findings to other socio-geographical contexts, particularly rural endemic areas, and restricts our ability to assess the long-term sustainability of the intervention effects. Future multi-center studies with longer follow-up are warranted to confirm these findings. Fourth, the use of a subjective, self-reported questionnaire may introduce the potential for social desirability bias, where participants might provide answers they believe are socially acceptable rather than reflecting their true knowledge or practices, although this is a common methodology in KAP studies [21,22]. Furthermore, a formal sample size calculation was not conducted a priori, as the study aimed to include all eligible students from the two participating schools, future studies would benefit from such a calculation to enhance statistical power.

## 5. Conclusions

In summary, this study provides robust evidence that peer-led education is a superior method for improving schistosomiasis-related knowledge among schoolchildren in a transmission-interrupted area of Wuhan, compared to routine health education. However, its advantage did not extend to attitudes and practices, highlighting the persistent challenge of translating knowledge into behavioral outcomes in populations with high baseline awareness. We recommend the strategic incorporation of peer-led modules to boost knowledge within school health initiatives. For enduring, comprehensive impact, future efforts should extend beyond the school walls to include community-wide engagement and environmental improvements, coupled with long-term evaluation to fully capture the evolution of the KAP trajectory.

## Supporting information

S1 Data Baseline & Post_intervention Questionnaire Response. (XLS)

S1 File Questionnaire in English. (DOC)

S2 File Questionnaire in Chinese. (DOC)
