# Supplementary material for: Peer-led versus routine health education for schistosomiasis knowledge improvement among primary school students in Wuhan, China
Source: PLoS Negl Trop Dis. 2026 Jan 2;20(1):e0013857. doi: 10.1371/journal.pntd.0013857 (PMC12758770; doi:10.1371/journal.pntd.0013857)
Supplement: S1 File — (DOC) [file pntd.0013857.s002.doc]

**Questionnaire on Knowledge, Attitudes, and Practices Regarding Schistosomiasis Prevention and Control**

This is a questionnaire survey on knowledge about schistosomiasis prevention and control. Please fill it out truthfully and provide your valuable feedback. This questionnaire is intended for scientific research only, to evaluate the latest education and awareness methods. It is anonymous, and we will keep all your personal information confidential. Please feel assured to fill it out.

Thank you very much for your participation and cooperation!

**Instructions: Please complete all sections.**

**Part 1: Basic Information**

*For the following items, please fill in the blanks or write your answer in the*

*space provided.*

**Gender** ( ) Male ( ) Female ***(Tick one box*)**

**Age**  Years

**School name**

**Grade&Class**  Grade Class

**Part 2: Knowledge on Schistosomiasis Prevention and Control**

*For the following multiple-choice questions, please select the correct answer and write its corresponding letter (e.g., A, B, C, D) in the parentheses after each question.*

**1、The primary species of schistosomiasis prevalent in China is （ ）**

A. Schistosoma mansoni

B. Schistosoma haematobium

C. Schistosoma japonicum

D. Don't know

**2、The snail species that transmits schistosomiasis is ( )**

A. Pipe snail

B. Oncomelania hupensis (Mud snail)

C. Vegetable snail

D. Don't know

**3、At what time of the year are children most likely to get schistosomiasis from playing in water? ( )**

A. November - February

B. March - April

C. April - October

D. Don't know

**4、One contracts schistosomiasis through ( )**

A. Eating contaminated food

B. Contact with infected individuals

C. Contact with water in snail-inhabited areas

D. Don't know

**5、The primary symptoms experienced after contracting schistosomiasis are ( )**

A. Accelerated heartbeat

B. Fever, abdominal pain, diarrhea

C. Pain in the hands and feet

D. Don't know

**6、Approximately how many days does it take from contacting waters with Oncomelania snails to getting sick? ( )**

A. Don't know

B. 7~15 days

C. 20 days

D. 40 days

**7、The first-line drug for the treatment of schistosomiasis is ( )**

A. Medicine for reducing fever

B. Medicine for treating diarrhea

C. Medicine specifically for killing parasites

D. Don't know

**8、Which medicine works best against schistosomiasis? ( )**

A. Ordinary lime

B. Bleaching powder

C. Specific molluscicides

D. Don't know

**9、What is mainly used now to eliminate Oncomelania snails? ( )**

A. Ordinary lime

B. Bleaching powder

C. Special molluscicide (snail-killing medicine)

D. Don't know

**10、After humans or livestock contract schistosomiasis, can their feces spread the disease when introduced into water? ( )**

A. Yes

B. No

C. Not sure

D. Don't know

**Part 3: Attitudes on Schistosomiasis Prevention and Control**

*For the following multiple-choice questions, please select the correct answer and write its corresponding letter (e.g., A, B, C, D) in the parentheses after each question.*

**11、If you suspect you have schistosomiasis, are you willing to seek testing and treatment? ( )**

A. Yes, I am. B. No, I am not.

**12、Do you believe that schistosomiasis is( )**

A. Preventable

B. Not preventable

C. Indifferent

**13、Do you believe that schistosomiasis can be eradicated? ( )**

A. Yes

B. No

C. Indifferent

**Part 4: Health Practices on Schistosomiasis Prevention and Control**

*For the following multiple-choice questions, please select the correct answer and write its corresponding letter (e.g., A, B, C, D) in the parentheses after each question.*

**14、In the past month, do you go to waters in areas with Oncomelania snails to wash rice, bathe, do laundry, or play in the water? ( )**

A. Occasionally

B. Frequently

C. Never

**15、In the past month, when entering waters in areas with Oncomelania snails, do you take protective measures such as wearing rubber boots, gloves, applying repellent, or using protective clothing? ( )**

A. Yes

B. No

**16．In the past month, when you suspect you have been exposed to infested water or infected with schistosomiasis, will you seek medical attention immediately? ( )**

A. Go immediately

B. Don't want to go

C. Don't know what to do
