# Supplementary material for: Peer-led versus routine health education for schistosomiasis knowledge improvement among primary school students in Wuhan, China
Source: PLoS Negl Trop Dis. 2026 Jan 2;20(1):e0013857. doi: 10.1371/journal.pntd.0013857 (PMC12758770; doi:10.1371/journal.pntd.0013857)
Supplement: S2 File — (DOC) [file pntd.0013857.s003.doc]

**血吸虫病防治知信行调查问卷**

您好：

感谢您参与此项调研。这是一份关于血吸虫病防治知识、态度和行为的问卷调查，请您如实填写，并提出宝贵意见。本问卷仅用做科学研究，为评价最新的宣传教育方式提供依据，此问卷为匿名填写，我们将对您的一切个人信息保密，请放心填写，谢谢合作！

**填写说明：请完整填写以下所有内容**。

**一、基本情况**

请直接在下划线上填写，或在相应括号内打“√”。

**性别：**男（ ） 女（ ）***（请勾选一项）***

**年龄：** 岁

**学校名称：**

**年级班级：** 年级 班

1. **血防知识**

请为下列单选题选出正确答案，并将其选项字母（如 A、B、C、D）填写在

题干的括号内。

1、在我国流行的血吸虫病主要是（ ）

A、曼氏血吸虫   B、埃及血吸虫   C、日本血吸虫   D、不知道

2、传播血吸虫病的螺类是（ ）

A、管螺   B、钉螺    C、菜螺    D、不知道

3、在一年中的哪个时候，小朋友因为玩水最容易得血吸虫病？（ ）

A、11月—2月    B、3月—4月  C、4月—10月 D、不知道

4、得血吸虫病是因为（ ）

A、吃不干净的食物  B、接触病人  C、到有螺地带下水  D、不知道

5、得了血吸虫病后主要感觉是（ ）

A、心跳加快 B、发烧、腹痛、腹泻  C、手脚病   D、不知道

6、从接触有钉螺的水体到生病，大概会经过多少天？（ ）

A、不知道 　B、7~15天　 C、20天　  D、40天

7、对付血吸虫，最有效的药物是？（ ）

A、帮助退烧的药 B、治疗腹泻的药 C、专门杀虫的药 　 D、不知道

8、现在消灭钉螺，主要使用的是（ ）

A、 普通的石灰 B、漂白用的粉末  C、专门的灭螺药物   D、不知道

9、最简单有效防止得血吸虫病的办法是（ ）

A、不接触病人   B、不接触粪便    C、不接触疫水 D、不知道

10、人、畜得血吸虫病后，它的粪便进入水中可以传播血吸虫病吗？（ ）

A、是      B、不是的    C、不确定 D、不知道

**三、血防态度**

请为下列单选题选出正确答案，并将其选项字母（如 A、B、C、D）填写在

题干的括号内。

11、当你怀疑得了血吸虫病，你愿意去检查、治疗吗？（ ）

A、愿意          B、不愿意

12、你觉得血吸虫病是（ ）

A、可以预防的   B、不可以预防的 C、无所谓

13、你认为血吸虫病是（ ）

A、能够消灭       B、无法消灭      C、无所谓

**四、卫生行为**

请为下列单选题选出正确答案，并将其选项字母（如 A、B、C、D）填写在

题干的括号内。

14、过去一个月内，你到不到有钉螺地带的水中淘米、洗澡、洗衣服、玩水（ ）

A、偶尔去      B、经常去      C、不去

15、过去一个月内，你在有钉螺的地带下水时，穿胶鞋、戴手套、涂药或穿防护衣吗？（ ）

A、是       B、不是的

16．过去一个月内，当你怀疑自己接触了疫水或者得了血吸虫病时，你会马上去看医生吗？（ ）

  A、马上去       B、不想去        C、不知道怎么办
